# Systems Toxicology Indicates AKT1/PTGS2‐Centered Neuroinflammatory‐Mitochondrial Crosstalk in Lidocaine Associated Neurotoxicity

**DOI:** 10.1002/cns.71218

**Published:** 2026-10-26

**Authors:** Min Jiang, Liping Chen, Jingyi Deng, Hangyu Li, Yuqia Xie, Xueqin Huang, Weidong Liu, Suidong Ouyang

**Affiliations:** ^1^ Department of Pediatrics, the First Dongguan Affiliated Hospital Guangdong Medical University Dongguan Guangdong China; ^2^ Dongguan Innovation Institute The First Dongguan Affiliated Hospital, Guangdong Medical University Dongguan Guangdong China; ^3^ Guangdong Provincial Key Laboratory of Medical Immunology and Molecular Diagnostics The First Dongguan Affiliated Hospital, Guangdong Medical University Dongguan China

**Keywords:** epilepsy, lidocaine, network toxicology, neurotoxicity, teething gel

## Abstract

**Background:**

Lidocaine‐containing teething gels are commonly used for pediatric oral analgesia, yet excessive exposure may induce severe neurotoxic effects, including seizure‐related events. The molecular mechanisms underlying lidocaine‐associated neurotoxicity remain poorly defined.

**Methods:**

We employed an integrative systems toxicology approach combining network toxicology, transcriptomic analysis (GSE143272), weighted gene co‐expression network analysis, molecular docking, molecular dynamics simulation, and experimental validation using Kamistad Gel extract at defined lidocaine‐equivalent concentration to characterize the molecular basis of lidocaine‐induced neurotoxicity.

**Results:**

Sixteen shared targets were identified through the integration of lidocaine‐related targets, epilepsy‐associated genes, and transcriptomic signatures. AKT1 and PTGS2 emerged as key hub genes. Functional analyses consistently suggested neuroinflammatory signaling, mitochondrial dysfunction, and synaptic dysregulation as major pathogenic mechanisms. Molecular docking and 10‐ns molecular dynamics simulations indicated stable interactions between lidocaine and AKT1/PTGS2. ROC analysis indicated moderate predictive performance of these genes in epilepsy datasets. In SH‐SY5Y cells, Kamistad Gel extract significantly increased AKT1 and PTGS2 expression, supporting activation of inflammation‐related neurotoxic pathways.

**Conclusions:**

Lidocaine‐associated neurotoxicity may involve an AKT1/PTGS2‐centered neuroinflammatory–mitochondrial crosstalk network accompanied by synaptic dysfunction. This study provides a systems‐level mechanistic insight into lidocaine neurotoxicity and identifies potential targets for improving the safety of pediatric lidocaine‐containing formulations.

## Graphical abstract

Lidocaine‐associated neurotoxicity is medicated by AKT1/PTG2 centered neuroinflammatory‐synaptic dysregulation coupled with mitochondrial impairment. This systems‐level insight provides a theoretical basis for improving the safety of pediatric lidocaine‐containing formulations.

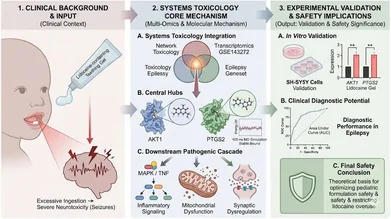

## 1 Introduction

Teething is a normal developmental process in infants and young children, but it is often accompanied by discomfort symptoms such as gingival irritation, irritability, drooling, sleep disturbance, and reduced appetite [1, 2, 3, 4]. To relieve these symptoms, lidocaine‐containing teething gels are widely used in pediatric practice because of their rapid and effective local anesthetic properties [5, 6]. In addition to commercially available products, individualized hospital‐prepared formulations are also commonly applied in children [7]. Compound Chamomile and Lidocaine Hydrochloride Gel (Kamistad Gel), which contains lidocaine, thymol, and chamomile, is frequently used to alleviate discomfort during primary tooth eruption [8]. Although chamomile and thymol have not been clearly associated with systemic toxicity, lidocaine itself is known to possess potential neurotoxic effects despite its clinical efficacy [9].

Children are particularly susceptible to lidocaine toxicity because of immature swallowing reflexes, limited dose‐control compliance, and developmental differences in drug metabolism [7, 10]. Excessive ingestion of lidocaine‐containing gels may rapidly elevate systemic drug concentrations, leading to severe neurological adverse events, including seizures, respiratory depression, and even death [10, 11]. More than 200 reported pediatric adverse events and the FDA black box warning have highlighted the clinical significance of this safety concern [7, 12, 13]. Furthermore, the poor dosing precision and rapid liquefaction of conventional gels may increase the risk of repeated administration and cumulative lidocaine exposure [6]. Although seizure‐related neurotoxicity associated with lidocaine exposure has been increasingly reported, its molecular mechanisms remain poorly understood because of the complex and heterogeneous nature of epilepsy [14, 15]. Emerging evidence indicates that neuroinflammation, mitochondrial dysfunction, and synaptic imbalance are key contributors to epileptogenic neurotoxicity. However, whether lidocaine‐induced neurotoxicity involves coordinated dysregulation of these processes remains unclear. Moreover, previous studies have largely focused on clinical manifestations and toxicokinetic characteristics, while systems‐level mechanistic investigations are still lacking.

Given that lidocaine is the key neurotoxic component of concern, computational analyses in this study focused on lidocaine, whereas experimental validation was performed using a lidocaine‐containing Kamistad Gel extract at defined lidocaine‐equivalent concentrations. Using an integrative systems toxicology framework [16], we integrated network toxicology, transcriptomic analysis, weighted gene co‐expression network analysis (WGCNA), molecular interaction modeling, and experimental validation, and identified AKT1 and PTGS2 as central regulatory hubs potentially involved in lidocaine‐induced neurotoxic responses. Functional analyses further supported that inflammatory signaling, mitochondrial dysfunction, and synaptic dysregulation may collectively contribute to the neurotoxic effects of lidocaine exposure. These findings provide a systems‐level mechanistic insight into lidocaine‐associated neurotoxicity and offer a theoretical basis for improving the safe clinical use of lidocaine‐containing formulations in pediatric populations.

## 2 Materials and Methods

### 2.1 Toxicological Prediction of Lidocaine

To align compound‐level predictions with formulation‐relevant validation, we preliminarily evaluated the neurotoxic potential of lidocaine. The canonical SMILES structure of lidocaine was submitted to ProTox‐3.0 and ADMETlab 3.0 for in silico toxicity prediction. Toxicity classification, blood–brain barrier permeability, and neurotoxicity‐related endpoints were analyzed. Integrated prediction results from both platforms were subsequently used to characterize the toxicological profile of lidocaine and guide downstream mechanistic analyses.

### 2.2 Transcriptomic Data Acquisition and Differential Expression Analysis

The epilepsy‐related transcriptomic dataset GSE143272, derived from peripheral blood samples of epilepsy patients, was obtained from the Gene Expression Omnibus (GEO) database. After quality control and removal of one duplicated sample (GSM4255857), 50 healthy controls and 34 untreated epilepsy samples were included for subsequent analyses. Probe IDs were converted into corresponding gene symbols according to platform annotation files, and the median expression value was retained for genes mapped by multiple probes. Principal component analysis (PCA) was performed on the normalized expression matrix to evaluate transcriptomic distribution patterns and intergroup variability between epilepsy and control samples. Differentially expressed genes (DEGs) were identified using the empirical Bayes method implemented in the limma package. Genes with |log_2_ fold change| > 0.15 and adjusted *p* < 0.05 were considered significantly differentially expressed. Volcano plots and hierarchical clustering heatmaps were generated to visualize global transcriptional alterations and expression patterns.

### 2.3 Weighted Gene co‐Expression Network Analysis (WGCNA)

To identify epilepsy‐associated co‐expression modules, the top 25% of genes ranked by expression variability from the GSE143272 dataset were subjected to WGCNA after removal of missing values and outlier samples. A weighted gene co‐expression network was constructed using the WGCNA R package. The optimal soft‐thresholding power (β) was determined according to the scale‐free topology criterion. Gene modules were identified using dynamic tree cutting with a minimum module size of 30 and a module merging threshold of 0.25. Module eigengenes were subsequently correlated with epilepsy phenotypes to identify disease‐associated modules. Gene significance (GS) and module membership (MM) values were calculated to evaluate module reliability. To comprehensively capture epilepsy‐related transcriptional signatures while minimizing potential target omission caused by disease heterogeneity, genes from key modules were integrated with DEGs to generate a consolidated candidate target set for downstream analyses.

### 2.4 Identification of Lidocaine and Epilepsy Associated Targets

Potential lidocaine‐associated targets were retrieved from multiple curated databases, including SwissTargetPrediction, SEA, STITCH, CTD, SuperPred, and PharmMapper. All targets were restricted to *Homo sapiens* and standardized using UniProt annotations. After duplicate removal, a unified lidocaine‐target dataset was established. Epilepsy‐associated genes were collected from GeneCards, OMIM, OpenTargets, and TTD databases. To improve disease specificity and reduce false‐positive associations, only targets with GeneCards relevance scores ≥ 5 and OpenTargets scores ≥ 0.1 were retained. After standardization and deduplication, an integrated epilepsy‐associated gene dataset was generated.

### 2.5 Construction of the Protein–Protein Interaction (PPI) Network

To identify potential mediators of lidocaine‐associated neurotoxicity, lidocaine‐related targets were intersected with epilepsy‐associated genes and transcriptome‐derived candidate genes obtained from DEG and WGCNA analyses. Overlapping genes were considered candidate targets potentially involved in lidocaine‐induced neurotoxic responses. These targets were imported into the STRING database ( *Homo sapiens* restricted) to construct a protein–protein interaction (PPI) network with a confidence score threshold of ≥ 0.4. Network visualization and subsequent analyses were performed using Cytoscape software.

### 2.6 Functional Enrichment Analysis

Gene Ontology (GO) and Kyoto Encyclopedia of Genes and Genomes (KEGG) enrichment analyses were conducted using the DAVID platform [17]. GO analysis included biological process (BP), cellular component (CC), and molecular function (MF) categories. KEGG analysis was performed to identify signaling pathways potentially associated with lidocaine‐induced neurotoxicity. Terms with *p* < 0.05 were considered significantly enriched. The top enriched GO terms and KEGG pathways were visualized using bar plots, bubble plots, and Sankey diagrams to illustrate functional interactions among candidate targets and neurotoxicity‐related pathways [18].

### 2.7 Gene Set Enrichment Analysis (GSEA)

To further validate pathway‐level alterations, Gene Set Enrichment Analysis (GSEA) was performed using the complete ranked gene list generated from differential expression analysis. Hallmark and curated gene sets obtained from the Molecular Signatures Database (MSigDB) were used as reference datasets. Analyses were conducted using the clusterProfiler and org.Hs.eg.db packages in R. Pathways with |NES| > 1, adjusted *p* < 0.05, and *q* < 0.25 were considered significantly enriched. Enrichment plots were generated using the enrichplot package to visualize pathway‐level transcriptional alterations associated with epilepsy‐related neurotoxicity.

### 2.8 Topological Identification of Hub Targets

The PPI network was analyzed in Cytoscape using the CytoHubba plugin [19]. To improve robustness and reduce algorithmic bias, six topological algorithms, including Degree, MNC, BottleNeck, Betweenness, Closeness, and Radiality, were applied to rank candidate targets. The top 10 genes from each algorithm were intersected to identify consensus hub genes potentially involved in lidocaine‐associated neurotoxicity.

### 2.9 Molecular Docking Analysis

Hub targets identified from topological analysis were subjected to molecular docking analysis [20]. High‐resolution crystal structures of target proteins were obtained from the RCSB Protein Data Bank (PDB). Protein structures were preprocessed using PyMOL 2.3.0 by removing water molecules and nonessential ions. Polar hydrogens and docking parameters were subsequently prepared using AutoDock Tools. The three‐dimensional structure of lidocaine was obtained from PubChem and energy‐minimized using Chem3D. Molecular docking simulations were performed using AutoDock Vina with 10 independent docking runs for each protein–ligand complex. Binding conformations with the lowest binding energy were selected for further interaction analysis and visualization using PyMOL and Discovery Studio. Binding energies ≤ −5 kcal/mol were considered indicative of stable interactions.

### 2.10 Molecular Dynamics Simulations

To further evaluate binding stability, molecular dynamics (MD) simulations of lidocaine‐AKT1 and lidocaine–PTGS2 complexes were performed using GROMACS v2022.3 [21, 22]. Ligand parameters were generated using the GAFF force field in AmberTools, while protein topologies were constructed using the Amber99SB‐ILDN force field. Complexes were solvated in a TIP3P water model with ion neutralization under physiological conditions. After energy minimization using the steepest descent algorithm, systems underwent NVT and NPT equilibration at 300 K, followed by 10‐ns production simulations with a 2‐fs integration step. Root mean square deviation (RMSD) and root mean square fluctuation (RMSF) analyses were performed to evaluate structural stability, ligand binding stability, and residue flexibility.

### 2.11 Receiver Operating Characteristic (ROC) Analysis

To evaluate the potential discriminatory performance of hub targets, ROC curve analysis was performed using the pROC package in R [23]. Expression levels of AKT1, PTGS2, and their combined predictive model were analyzed using epilepsy and control samples from the GSE143272 dataset. The area under the ROC curve (AUC) and 95% confidence intervals were calculated to assess predictive performance.

### 2.12 Cell Viability Assay

Human SH‐SY5Y neuroblastoma cells were cultured in a 1:1 mixture of MEM and F‐12 medium supplemented with 10% fetal bovine serum (FBS). Kamistad Gel extract (STADA Arzneimittel AG, Germany) was prepared at different concentrations based on the corresponding lidocaine‐equivalent concentrations and used for cytotoxicity assessment. Cells were seeded into 96‐well plates at a density of 5 × 10^4^ cells/well and maintained at 37°C in a humidified atmosphere containing 5% CO_2_. After 24 h of incubation, cell viability was determined using the Cell Counting Kit‐8 (CCK‐8) assay (Beyotime, China) according to the manufacturer's instructions. Each experiment was performed in technical triplicate, and at least three independent biological replicates were conducted.

### 2.13 RT‐qPCR

Total RNA was extracted from SH‐SY5Y cells using TRIzol reagent and reverse‐transcribed into cDNA using a commercial reverse transcription kit. Quantitative PCR was performed using SYBR Green Master Mix with gene‐specific primers for *Akt1*, *Ptgs2*, and *Gapdh* (Table 1). Relative mRNA expression levels were calculated using the 2^−ΔΔCt^ method and normalized to *Gapdh* expression. Each experiment was independently performed in triplicate.

**TABLE 1:** Primers used for RT‐qPCR.

Gene	Forward primer (5′‐3′)	Reverse primer (5′‐3′)
*Akt1*	TCCTCCTCAAGAATGATGGCA	GTGCGTTCGATGACAGTGGT
*Ptgs2*	TAAGTGCGATTGTACCCGGAC	TTTGTAGCCATAGTCAGCATTGT
*Gadph*	GGAGCGAGATCCCTCCAAAAT	GGCTGTTGTCATACTTCTCATGG

### 2.14 Reactive Oxygen Species (ROS) Measurement

ROS levels were assessed using a DCFH‐DA–based ROS assay kit (Beyotime, China) and flow cytometry. After treatment with Kamistad Gel extract for 24 h, SH‐SY5Y cells were washed with PBS, trypsinized, and centrifuged at 400 × *g* for 5 min. Cells were resuspended in serum‐free medium at 1 × 10^6^–2 × 10^6^ cells/mL and incubated with 10 μmol/L DCFH‐DA for 20 min at 37°C in the dark, with gentle mixing every 3 min. Cells were then washed three times with serum‐free medium, resuspended in pre‐chilled PBS, and analyzed by flow cytometry within 30 min while protected from light. ROS levels were quantified as the mean fluorescence intensity (MFI) of the DCFH‐DA signal and normalized to the corresponding control group.

### 2.15 Intracellular ATP Measurement

Intracellular ATP levels were determined using an ATP assay kit (HY‐K0314, MCE, USA) according to the manufacturer's instructions. Following treatment with Kamistad Gel extract for 24 h, SH‐SY5Y cells were lysed with 200 μL of lysis buffer per well, and the lysates were centrifuged at 12,000 × *g* for 5 min at 4°C. The supernatants were collected for ATP analysis. An ATP standard curve was prepared using standards ranging from 0.01 to 5 μmol/L. Briefly, 100 μL of ATP detection working solution was added to an opaque 96‐well plate and preincubated at room temperature for 3–5 min, followed by addition of 10 μL of each sample or standard. Chemiluminescence was immediately measured and expressed as relative light units (RLU). ATP concentrations were calculated from the standard curve.

### 2.16 Western Blot Analysis

Total protein was extracted from SH‐SY5Y cells using RIPA lysis buffer supplemented with 1% PMSF and a protease inhibitor cocktail (Roche, USA) on ice for 30 min. Cell lysates were centrifuged at 12,000 × *g* for 20 min at 4°C, and the supernatants were collected. Protein concentrations were determined using a BCA protein assay, and equal amounts of protein (20 μg per sample) were separated by 10% SDS‐PAGE and transferred onto PVDF membranes (Millipore, USA). After blocking, the membranes were incubated overnight at 4°C with primary antibodies against PTGS2 (Proteintech, Cat. No. 66351–1‐Ig), AKT (Cell Signaling Technology, Cat. No. 4685), phosphorylated AKT (Cell Signaling Technology, Cat. No. 2965), and GAPDH (Cell Signaling Technology, Cat. No. 2118S). After incubation with the corresponding HRP‐conjugated secondary antibodies, protein bands were visualized using an enhanced chemiluminescence (ECL) substrate (Millipore, USA) and imaged with an Amersham AI600 imaging system (GE Healthcare, USA).

### 2.17 Statistical Analysis

Statistical analyses were performed using GraphPad Prism and SPSS software. Data are presented as mean ± standard deviation (SD). Comparisons between two groups were conducted using Student's *t*‐test, while multiple‐group comparisons were analyzed using ANOVA. A two‐sided *p* < 0.05 was considered statistically significant.

## 3 Results

### 3.1 Integrated Systems Toxicology Prioritizes an AKT1/PTGS2‐Centered Neuroinflammatory Network Underlying Lidocaine‐Associated Neurotoxicity

To systematically elucidate the molecular basis of lidocaine‐associated neurotoxicity, an integrative systems toxicology framework combining toxicological prediction, epilepsy transcriptomics, and network pharmacology analyses was established (Figure 1A). In silico toxicity assessment using ProTox‐3.0 and ADMETlab 3.0 consistently predicted a high neurotoxic liability for lidocaine, with neurotoxicity probabilities exceeding 0.90 and a predicted LD_50_ of 220 mg/kg, suggesting a potential association with seizure‐related neurological injury.

**FIGURE 1:**
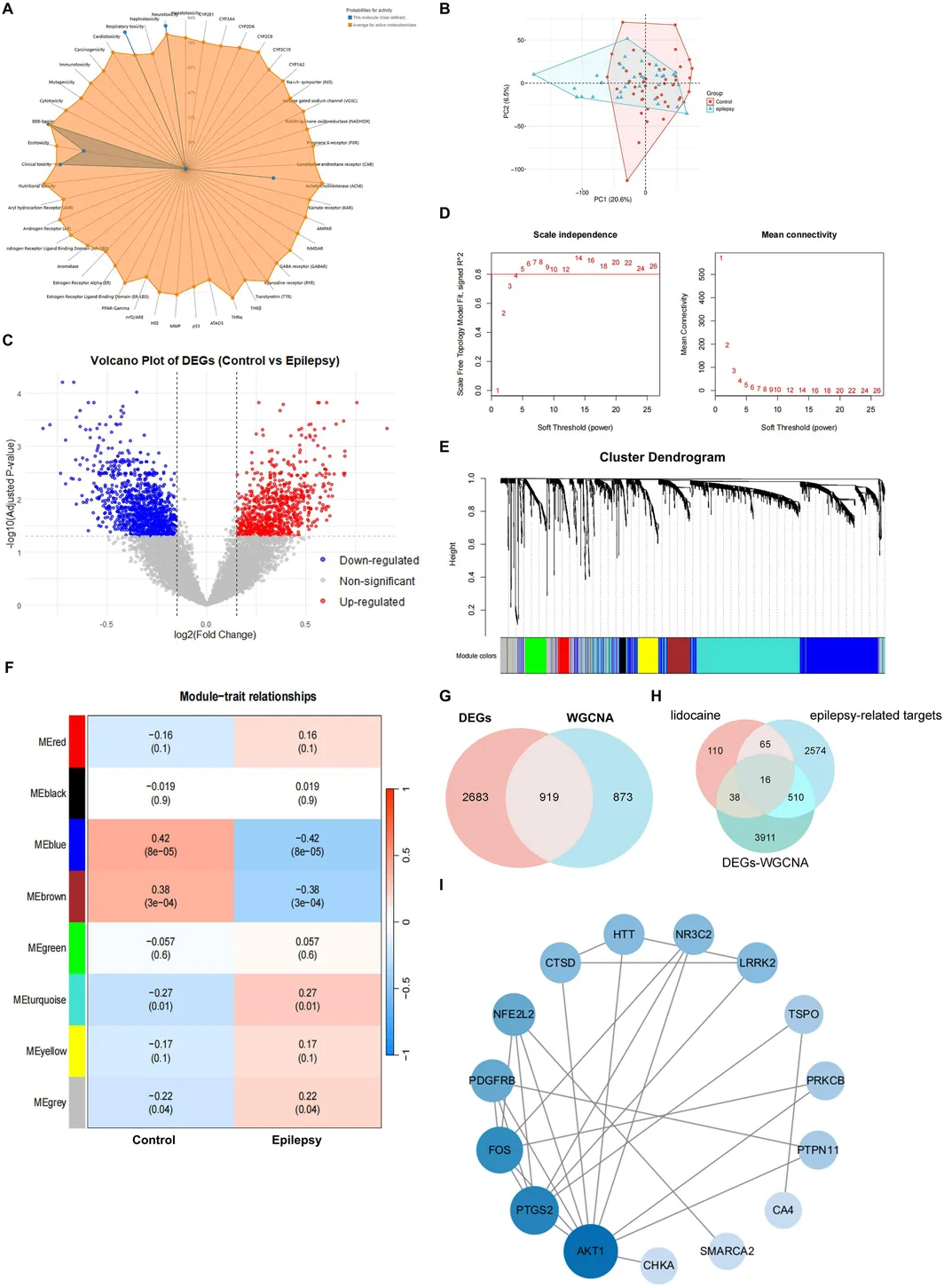
Integrated systems toxicology suggests AKT1/PTGS2 as potential hubs of lidocaine‐associated neurotoxicity. (A) Radar plot summarizing the predicted toxicity spectrum of lidocaine. The radial axis represents the probability scores for distinct toxicological endpoints, including neurotoxicity, respiratory toxicity, blood–brain barrier penetration, and clinical toxicity. The shaded region illustrates the overall predicted toxicity profile of lidocaine. (B) PCA plots of the GSE143272 dataset showing transcriptomic separation between control (orange) and epilepsy (blue) groups. (C) Volcano plot of DEGs. Red indicates upregulated genes, blue indicates downregulated genes, and gray indicates non‐significant genes. (D) Selection of the optimal soft‐thresholding power for WGCNA. The left panel shows the scale‐free topology fit index (R^2^), and the right panel shows mean connectivity. (E) Gene clustering dendrogram with co‐expression modules identified by dynamic tree cutting. (F) Heatmap of module–trait relationships. Red indicates positive correlations and blue indicates negative correlations. Values represent correlation coefficients and corresponding *p* values. (G) Venn diagram showing the overlap between DEGs and WGCNA derived hub genes, identifying epilepsy‐associated neurotoxic modules. (H) Venn diagram showing the overlap between lidocaine‐related targets and epilepsy‐associated genes. (I) Cytoscape visualization of the PPI network highlighting hub genes, including AKT1, PTGS2, and FOS. Larger and darker nodes indicate higher centrality.

To define disease‐relevant molecular signatures, the epilepsy transcriptomic dataset GSE143272 was analyzed. Principal component analysis demonstrated a clear separation between epilepsy and control samples (Figure 1B), indicating distinct transcriptional landscapes. Differential expression analysis identified 3602 dysregulated genes, including 1534 upregulated and 2068 downregulated genes (Figure 1C). WGCNA further identified three epilepsy‐associated modules (blue, brown, and turquoise) exhibiting the strongest correlations with disease status (Figure 1D–F). Integration of module genes and differentially expressed genes generated a comprehensive set of 4475 epilepsy‐related candidate genes (Figure 1G).

To identify molecular mediators linking lidocaine exposure to epilepsy‐associated neurotoxicity, 229 predicted lidocaine targets were intersected with 3168 epilepsy‐related genes. Sixteen overlapping targets were obtained and assembled into a highly interconnected protein–protein interaction network (Figure 1H,I). Network topology analysis consistently prioritized AKT1 and PTGS2 as the most influential hub nodes, with both genes ranking among the top two across topological algorithms (Figure S1 and Table S1). These findings point to an AKT1/PTGS2‐centered regulatory axis as a potential molecular bridge connecting lidocaine exposure to seizure‐associated neurotoxic responses.

### 3.2 Functional Analyses Indicate AKT1/PTGS2‐Associated Neuroinflammatory–Mitochondrial Convergence

To elucidate the biological processes governed by the identified target network, GO, KEGG, and GSEA analyses were performed. GO enrichment analysis demonstrated significant enrichment of pathways associated with kinase signaling, oxidative stress responses, inflammatory regulation, and cellular responses to xenobiotic stimuli, with the cellular components including presynaptic cytosol and intracellular signaling compartments (Figure 2A). KEGG pathway analysis further highlighted classical inflammatory cascades (particularly the MAPK and TNF signaling), alongside synaptic transmission pathways like cholinergic and dopaminergic synapses (Figure 2B). To further validate these findings at the transcriptomic level, GSEA independently corroborated the involvement of inflammatory axes involving CCR2/GNB/PI3K/NF‐κB signaling and additionally uncovered mitochondrial dysfunction‐related signatures, including TDP43‐associated electron transport impairment and UCP1‐mediated thermogenesis dysregulation (Figure 2C–F).

**FIGURE 2:**
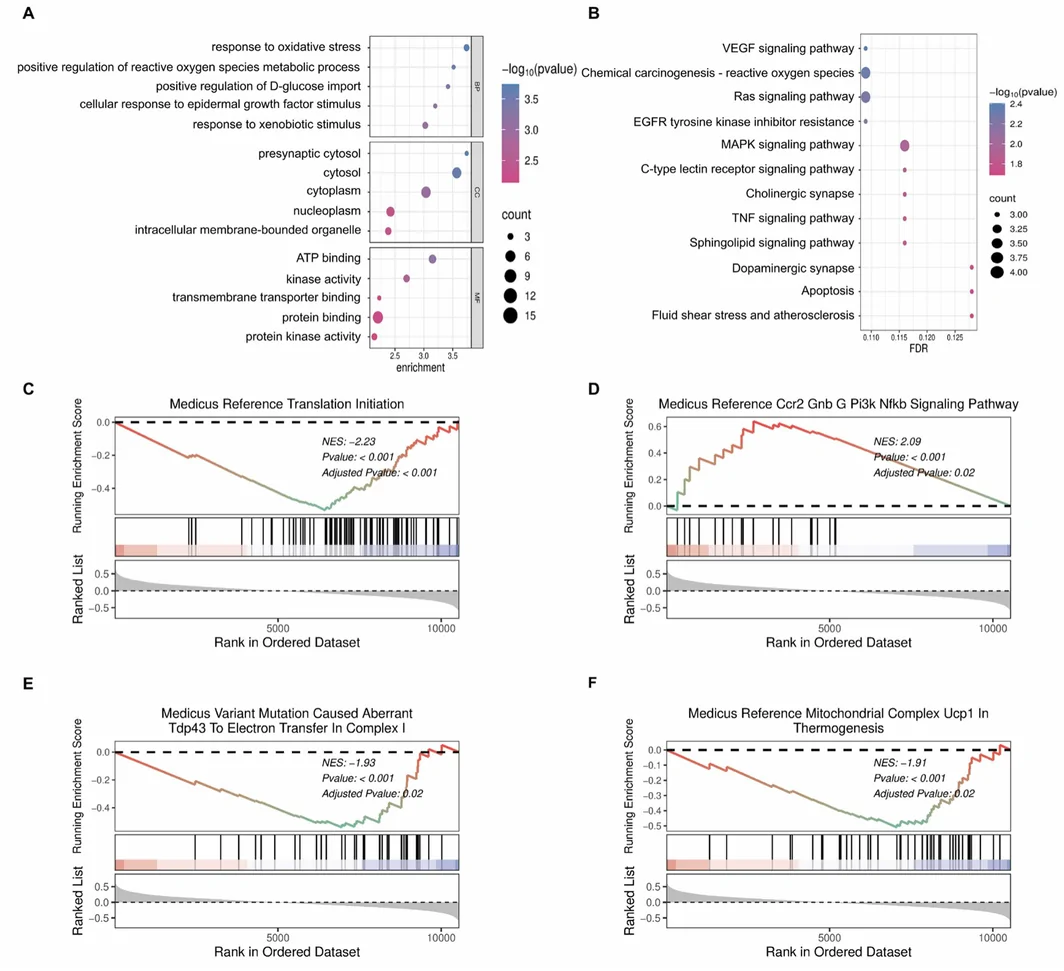
Functional enrichment is associated with inflammatory‐mitochondrial‐synaptic dysregulation. (A) GO enrichment analyses (BP, CC, and MF) presented as bubble plots. Bubble size represents gene count, and color intensity indicates enrichment significance. (B) Bubble plot of KEGG pathway enrichment analysis. Bubble size indicates gene count, and color intensity reflects enrichment significance. (C–F) Representative GSEA enrichment plots of pathways related to translation initiation, CCR2/GNB/PI3K/NF‐κB inflammatory signaling, mitochondrial electron transport dysfunction, and UCP1‐associated thermogenesis. Red lines indicate running enrichment scores.

These multi‐level enrichment results suggest a functional interplay between inflammatory and mitochondrial pathways, rather than independent events. The co‐enrichment of inflammatory signaling (MAPK/TNF, PI3K/NF‐κB) with mitochondrial dysfunction signatures (complex I impairment, UCP1 dysregulation) and synaptic pathways (cholinergic/dopaminergic synapses) implies a potential hierarchical connectivity, wherein inflammatory signals may link to metabolic disturbances that in turn affect synaptic function. Importantly, AKT1 and PTGS2 occupied central positions within these interconnected inflammatory and neuronal regulatory networks. Thus, these integrated analyses support a mechanistic framework in which AKT1/PTGS2‐associated neuroinflammatory signaling may converge with mitochondrial dysfunction and synaptic instability, potentially representing a pathogenic axis underlying lidocaine‐associated neurotoxicity.

### 3.3 Molecular Interaction Modeling Predicts Stable Binding of AKT1 and PTGS2 by Lidocaine

Given the central roles of AKT1 and PTGS2 within the identified neurotoxic network, molecular docking and MD simulations were conducted to evaluate their potential interactions with lidocaine. Docking analysis indicated favorable binding affinities between lidocaine and all prioritized hub proteins, with binding energies consistently below −5 kcal/mol (Table 2). Among them, AKT1 and PTGS2 displayed the most favorable binding conformations. Lidocaine was predicted to occupy well‐defined binding pockets and form multiple hydrogen‐bonding and hydrophobic interactions with key residues of both proteins (Figure 3A,B).

**TABLE 2:** Molecular docking results (binding energy).

No.	Ligand	Target protein	PDB ID	Binding energy (kcal/mol)
1	Lidocaine	AKT1	3CQU	−7.2
2	Lidocaine	PTGS2	5F19	−7.6
3	Lidocaine	FOS	1S9K	−5.8
4	Lidocaine	NFE2L2	2FLU	−6.8
5	Lidocaine	PDGFRB	3MJG	−6.4
6	Lidocaine	LRRK2	2ZEJ	−7.2
7	Lidocaine	PRKCB	8ES3	−6.4

**FIGURE 3:**
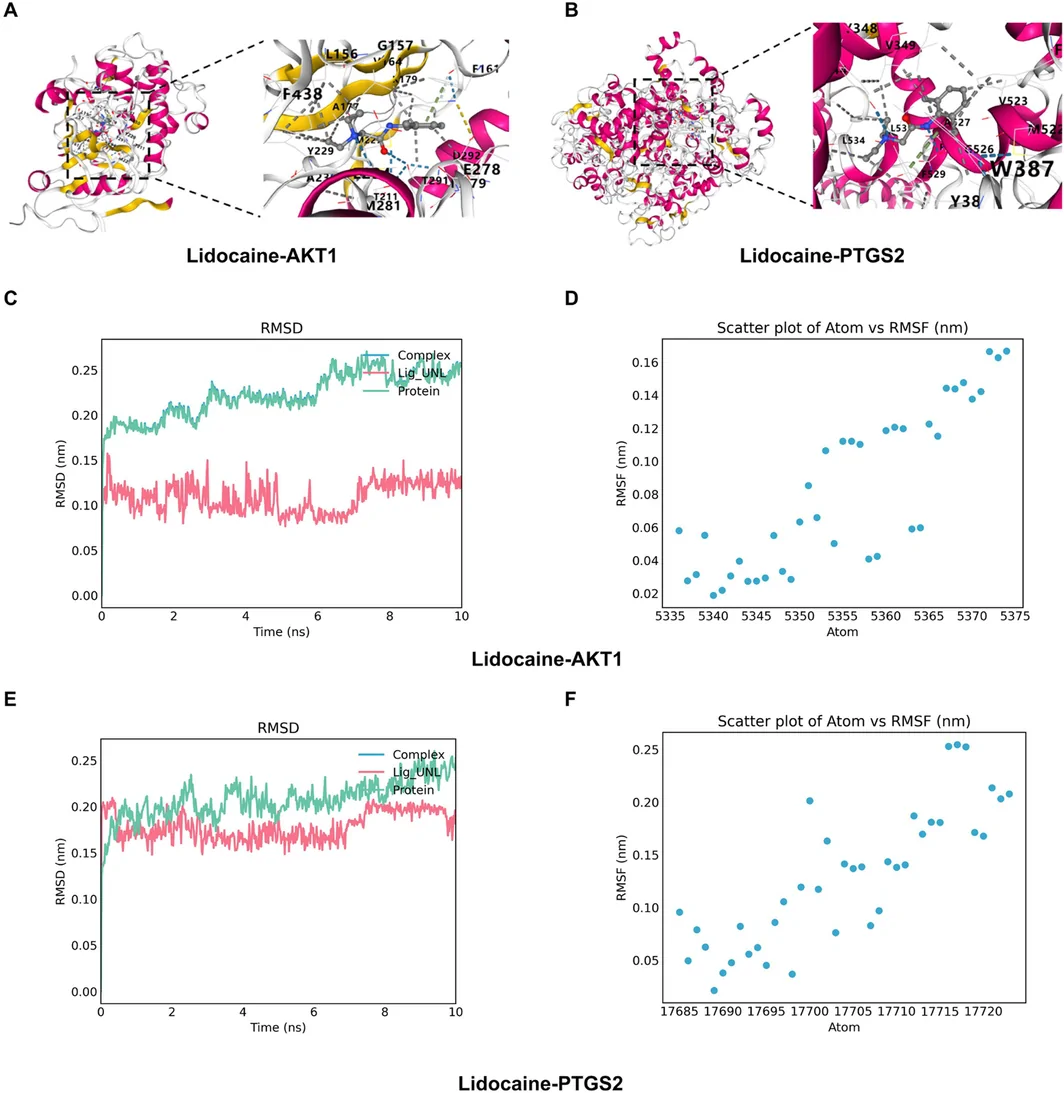
Molecular docking and MD simulation analyses of lidocaine‐AKT1 and lidocaine‐PTGS2 interactions. (A, B) Visualization of molecular docking analysis of lidocaine‐AKT1 and lidocaine‐PTGS2 complexes. (C, E) RMSD analyses of lidocaine–AKT1 and lidocaine–PTGS2 complexes during molecular dynamics simulations, showing the temporal stability of the protein–ligand complexes during the simulations. (D, F) RMSF analyses of lidocaine–AKT1 and lidocaine–PTGS2 complexes, showing residue flexibility and stable binding interfaces within ligand‐interacting regions.

To evaluate interaction stability under dynamic conditions, 10‐ns MD simulations were performed. RMSD trajectories demonstrated rapid equilibration within approximately 2 ns, followed by stable conformational behavior throughout the simulation period (Figure 3C,E). Consistently, RMSF analysis displayed minimal fluctuations within ligand‐binding regions, whereas greater flexibility was restricted to terminal and loop regions (Figure 3D,F). These findings suggest that lidocaine can stably interact with AKT1 and PTGS2, supporting their potential roles in linking neuroinflammatory and mitochondrial signaling disturbances in lidocaine exposure.

### 3.4 AKT1 and PTGS2 Show Moderate Discriminatory Performance in an Epilepsy‐Associated Transcriptomic Dataset

To preliminarily assess the potential relevance of the identified hub genes, ROC curve analysis was performed using the GSE143272 peripheral‐blood dataset. AKT1 demonstrated a moderate discriminative ability with an AUC of 0.685 (95% CI: 0.57–0.799), while PTGS2 showed a comparable performance with an AUC of 0.651 (95% CI: 0.525–0.776) (Figure 4). Although each gene alone provided limited classification power, their combined model achieved an AUC of 0.771 (95% CI: 0.663–0.879). These findings indicate that AKT1 and PTGS2 show moderate discriminatory performance in an epilepsy‐associated transcriptomic dataset. However, these ROC results should be interpreted as hypothesis‐generating rather than diagnostic validation for lidocaine neurotoxicity, given the non‐neural origin of this dataset.

**FIGURE 4:**
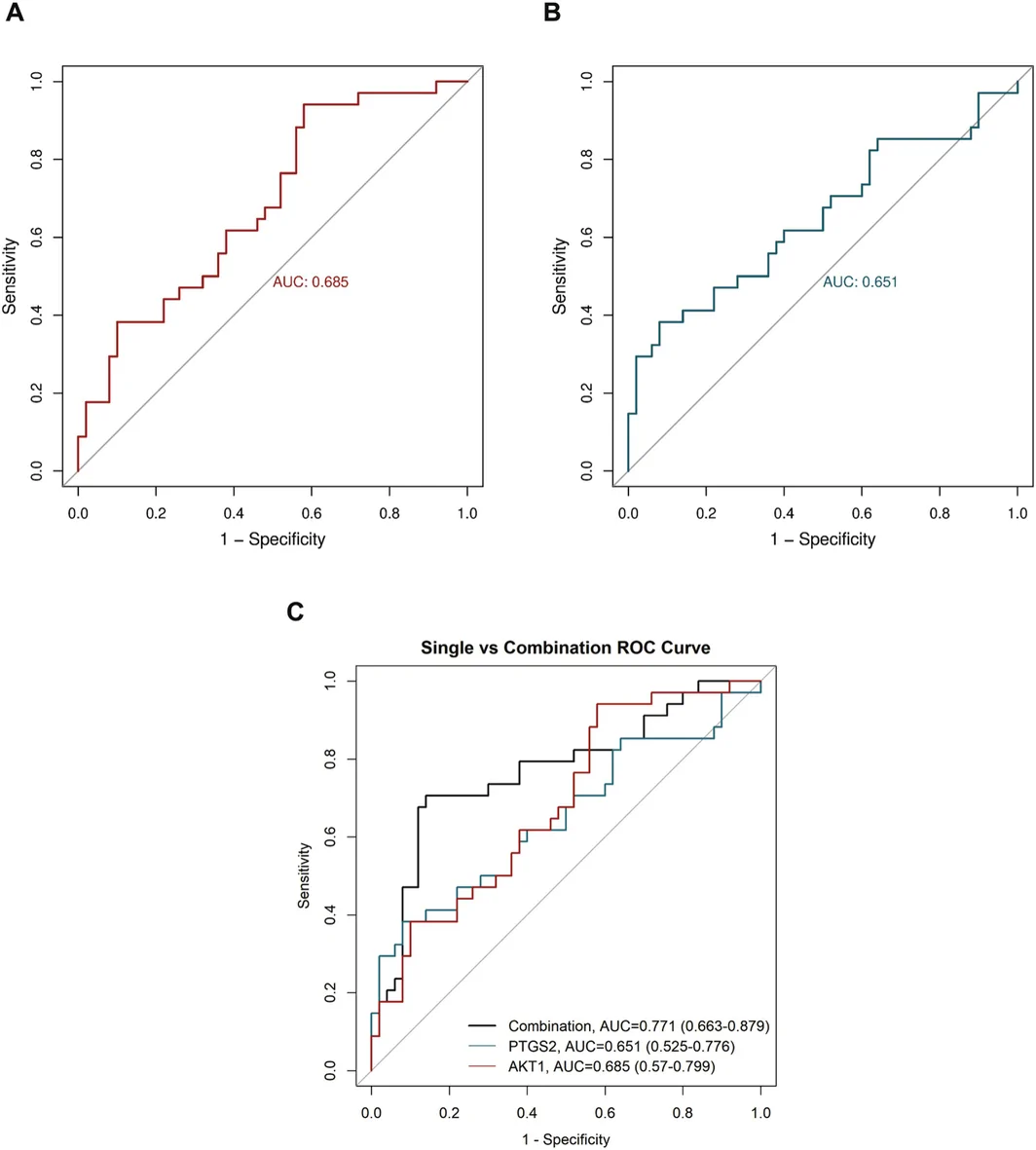
Discriminatory performance of AKT1 and PTGS2 in an epilepsy‐associated transcriptomic dataset. (A, B) ROC curve analyses of AKT1 and PTGS2 for evaluating epilepsy‐associated neurotoxic alterations. (C) ROC analysis of the combined AKT1/PTGS2 model indicating improved discriminatory performance compared with single‐gene models. Red, blue, and black curves represent AKT1, PTGS2, and the combined model, respectively.

### 3.5 Kamistad Gel Extract Increases AKT1 and PTGS2 Expression and Is Associated With Oxidative Stress and Reduced ATP Production in Neuronal Cells

To experimentally evaluate the key predictions generated by the systems toxicology analysis, SH‐SY5Y neuroblastoma cells were exposed to increasing concentrations of Kamistad Gel extract for 24 h. CCK‐8 assays demonstrated a concentration‐dependent reduction in cell viability (Figure 5A). Based on the observed cytotoxicity profile, 0.74 mM lidocaine‐equivalent exposure was selected for subsequent experiments. At this concentration, cell viability remained above 80%, indicating that the treatment caused measurable but limited cytotoxicity. RT‐qPCR and Western blot analyses showed increased AKT1 and PTGS2 expression following treatment compared with the control group (Figure 5B,C), consistent with the computational prioritization of these genes.

**FIGURE 5:**
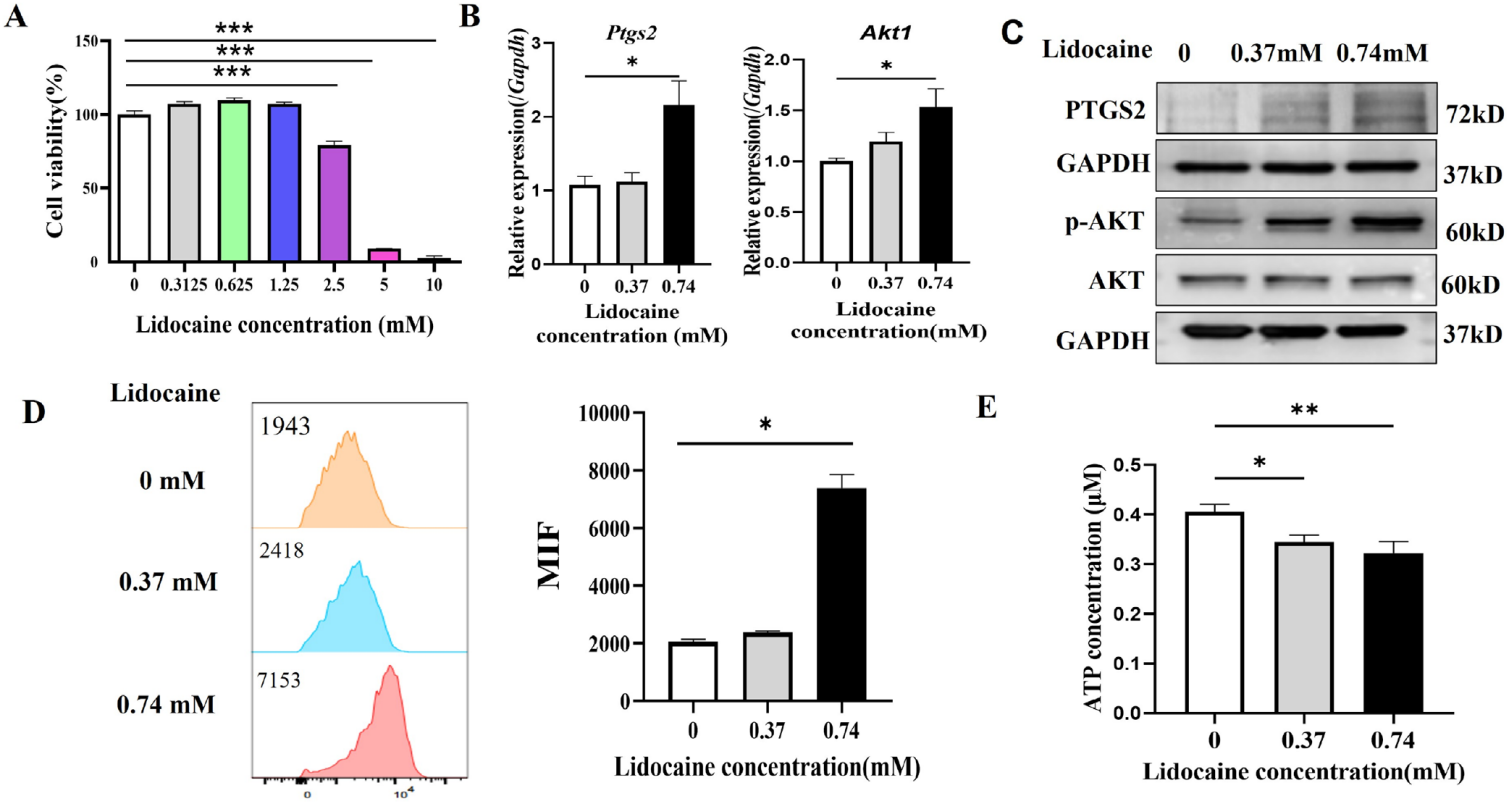
Experimental validation of systems toxicology predictions in Kamistad Geel extract‐treated neuronal cells. (A) Cell viability of SH‐SY5Y cells following 24 h exposure to increasing concentrations of lidocaine, as determined by the CCK‐8 assay. (B) Relative mRNA expression levels of AKT1 and PTGS2 in SH‐SY5Y cells following 24 h lidocaine exposure, as determined by RT‐qPCR. (C) Representative Western blot images and quantitative analysis of AKT1 and PTGS2 protein expression in SH‐SY5Y cells following lidocaine exposure. (D) Intracellular ROS levels in SH‐SY5Y cells following lidocaine exposure, as assessed by flow cytometry. (E) Cellular ATP levels following lidocaine exposure, as measured using an ATP assay kit. *p* < 0.05 and *p < 0.01 versus the corresponding control group.

We next examined cellular oxidative stress and energy metabolism. Flow cytometric analysis using DCFH‐DA showed a significant increase in intracellular ROS levels following treatment, whereas intracellular ATP levels were significantly reduced (Figure 5D,E). Together, these findings indicate that Kamistad Gel extract exposure is associated with increased oxidative stress and impaired cellular energy status in SH‐SY5Y cells. These experimental observations are consistent with the inflammatory and metabolic signatures identified by systems‐level analyses. However, the present data demonstrate associations rather than a direct causal relationship between AKT1/PTGS2 dysregulation and mitochondrial or metabolic dysfunction.

### 3.6 Proposed Mechanism of Lidocaine‐Associated Neurotoxicity Mediated by AKT1/PTGS2‐Centered Neuroinflammatory‐Mitochondrial Crosstalk

Based on the integration of network toxicology, transcriptomic analyses, molecular interaction modeling, and experimental validation, a mechanistic model was proposed (Figure 6). In this model, lidocaine is predicted to target AKT1 and PTGS2, leading to coordinated activation of neuroinflammatory pathways and mitochondrial dysfunction. The resulting neuroinflammatory–mitochondrial convergence disrupts synaptic homeostasis and neuronal function, thereby promoting neurotoxic responses. These findings identify the AKT1/PTGS2 axis as a potential mechanistic hub linking lidocaine exposure to seizure‐associated neuronal injury and provide systems‐level insights into the molecular basis of lidocaine‐induced neurotoxicity.

**FIGURE 6:**
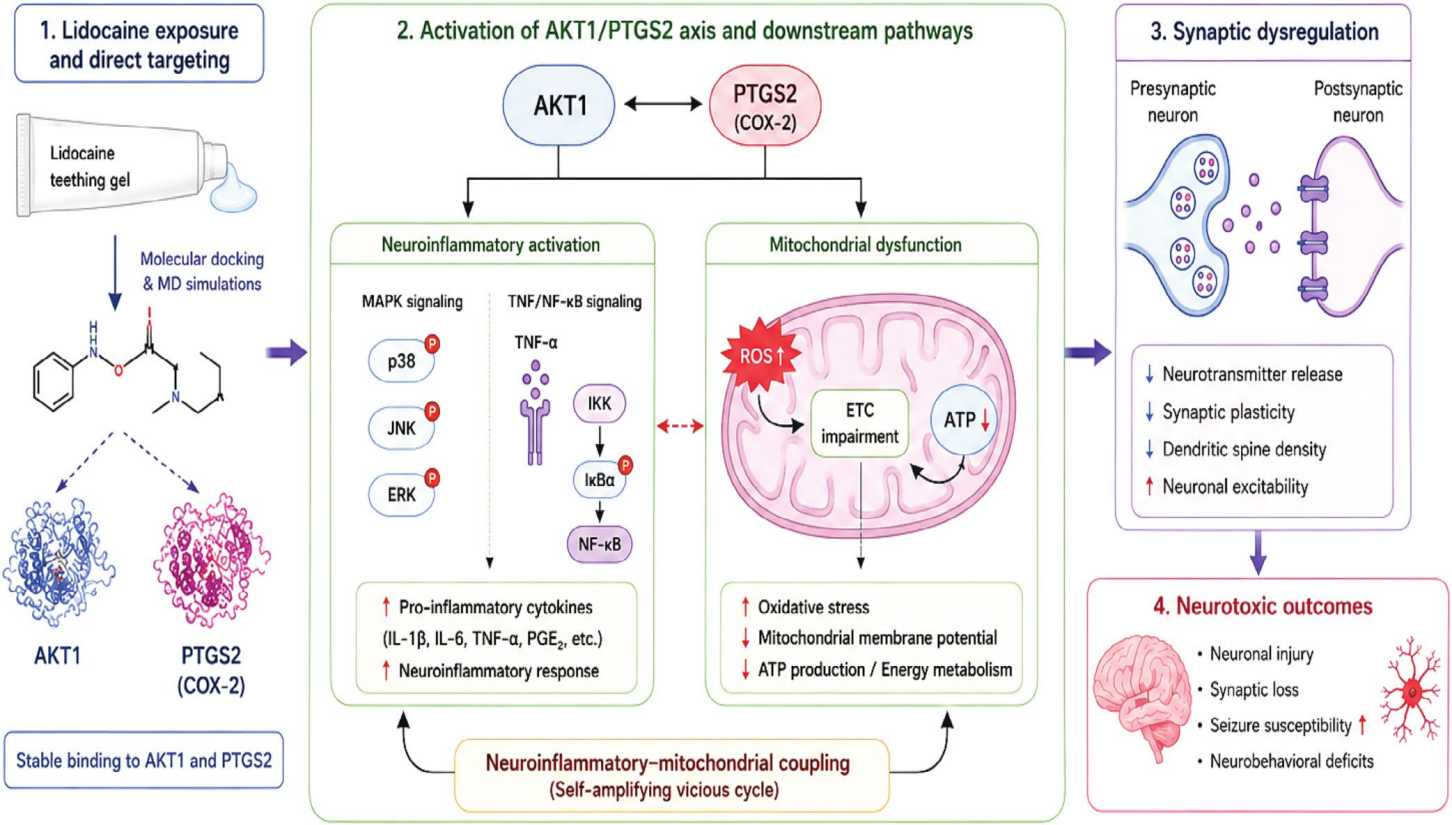
Proposed mechanistic model of lidocaine‐associated neurotoxicity. Lidocaine is predicted to interact with AKT1 and PTGS2, potentially influencing neuroinflammatory signaling and mitochondrial dysfunction. Activation of the AKT1/PTGS2 axis promotes MAPK/TNF/NF‐κB‐mediated inflammatory responses, ROS accumulation, impaired mitochondrial bioenergetics, and synaptic dysregulation. The resulting neuroinflammatory–mitochondrial crosstalk is related to neuronal injury and increases susceptibility to seizure‐associated neurotoxicity, highlighting AKT1/PTGS2 as a central pathogenic hub in lidocaine‐induced neural damage.

## 4 Discussion

Lidocaine‐containing teething gels are still widely used in pediatric oral analgesia despite repeated FDA warnings regarding seizure‐related neurotoxicity in infants and young children [13]. However, the molecular mechanisms underlying lidocaine‐associated neurotoxicity remain poorly defined. In this study, we integrated systems toxicology, transcriptomics, WGCNA, molecular interaction modeling, and experimental validation to investigate the molecular basis of lidocaine‐related neurotoxicity using an integrated systems toxicology framework. Our findings identified AKT1 and PTGS2 as central regulatory hubs linking inflammatory activation, synaptic dysregulation, and mitochondrial dysfunction. The predicted interactions and transcriptional responses were consistently observed in both computational and experimental validation. Taken together, these findings support a mechanistic model in which lidocaine‐associated neurotoxicity may involve AKT1/PTGS2‐mediated inflammatory‐synaptic dysregulation converged with mitochondrial impairment.

Neuroinflammation is increasingly recognized as a core driver of epileptogenesis [24, 25, 26, 27]. PTGS2, a key inducible enzyme in prostaglandin synthesis, is consistently elevated in epilepsy patients and seizure models [24, 25, 26, 27]. Increased PTGS2 activity promotes prostaglandin E2 production, enhances neuronal excitability, and lowers seizure threshold [28, 29, 30]. In our study, PTGS2 ranked second highest score in network prioritization. MAPK and TNF signaling pathways were significantly enriched, both of which are known to activate PTGS2‐associated inflammatory response, supporting PTGS2 as a downstream component of lidocaine‐induced inflammatory cascade [28, 31, 32, 33]. Consistent with these findings, PTGS2 expression was significantly increased in SH‐SY5Y cells following lidocaine‐containing gel exposure. Molecular docking and dynamics analyses further suggested stable lidocaine‐PTGS2 interactions, which may reflect an additional modulatory effect on inflammatory signaling beyond classical sodium‐channel blockade.

In addition to PTGS2, AKT1 was identified as the top hub gene in our topological identification. As a core regulator of the PI3K/AKT/mTOR pathway, AKT1 controls neuronal survival, synaptic plasticity, and excitatory‐inhibitory balance [34, 35, 36]. Dysregulation of this pathway has been strongly associated with neuronal hyperexcitability and epileptogenesis [37]. In RT‐qPCR validation, AKT1 showed significant upregulation in Kamistad Gel extract‐exposed SH‐SY5Y cells. Molecular simulations suggested stable lidocaine‐AKT1 binding, raising the possibility that lidocaine may affect AKT signaling. In parallel, our enrichment analyses also pointed to PI3K/NF‐κB‐related inflammatory pathways, supporting functional convergence between AKT1 activation and inflammatory signaling. This observation is notable because NF‐κB serves as a common node that can simultaneously regulate PTGS2 expression and PI3K/AKT pathway activity [28]. Thus, coordinated dysregulation of AKT1 and PTGS2 may represent a convergent mechanism underlying lidocaine‐associated neurotoxicity.

To further characterize the functional network surrounding AKT1 and PTGS2, we performed systematic enrichment analyses across GO, KEGG and GSEA platforms. Consistent with the oxidative stress and kinase signaling terms in GO analysis, KEGG pathways highlighted MAPK/TNF inflammatory pathways and synaptic signaling pathways, both of which are closely associated with epilepsy pathogenesis [38, 39, 40]. GSEA further identified CCR2‐mediated inflammatory signaling, mitochondrial complex I dysfunction, and UCP1‐associated metabolic abnormalities. These findings consistently suggest that mitochondria‐related transcriptional alterations may represent a potential molecular link between inflammatory signaling and synaptic dysregulation in lidocaine‐associated neurotoxicity. Specifically, impaired mitochondrial respiration can increase reactive oxygen species production, activate NF‐κB signaling, and further enhance PTGS2‐mediated inflammatory responses, potentially forming a positive feedback loop that promotes neuronal hyperexcitability and lowers seizure threshold [39, 41]. The convergence of the above analyses therefore supports a multidimensional pathogenic model involving inflammatory‐mitochondrial‐synaptic dysregulation. To explore the translational relevance of this model, we assessed the potential utility of AKT1 and PTGS2. The combined ROC analysis displayed improved discriminatory performance for AKT1/PTGS2 compared with either marker alone (AUC = 0.771), suggesting potential value as composite indicators associated with epilepsy‐related neurotoxic responses. Although the predictive performance remains moderate, these findings indicate that multi‐target molecular signatures may better reflect the complex nature of neurotoxicity than single‐gene indicators.

Several limitations should be acknowledged. First, transcriptomic analyses were based primarily on a single peripheral‐blood GEO dataset with a small sample size, and potential biases may be introduced by parameter thresholds in computational modeling (e.g., WGCNA soft‐thresholding, in silico toxicity models), suggesting these findings require validation in neural tissue‐derived or larger independent cohorts. Second, the SH‐SY5Y cell model and acute exposure conditions cannot fully reproduce the in vivo neuroimmune microenvironment or chronic epileptogenic processes. Third, although mRNA and protein‐level changes of AKT1 and PTGS2 were assessed, functional activity and downstream signaling remain incompletely explored. Finally, direct biochemical validation of lidocaine‐AKT1/PTGS2 interactions and in vivo seizure‐related studies are still needed.

## 5 Conclusion

This study proposed a systems‐level mechanistic framework for understanding lidocaine‐associated neurotoxicity. Integrative analyses suggested that AKT1 and PTGS2 may represent potential molecular hubs linking inflammatory signaling with mitochondrial and synaptic dysregulation in lidocaine‐associated epilepsy. Experimental validation using a lidocaine‐containing Kamistad Gel extract at a defined lidocaine‐equivalent exposure showed increased AKT1 and PTGS2 expression and was accompanied by increased ROS and reduced ATP levels in SH‐SY5Y cells, consistent with the proposed inflammatory–mitochondrial mechanism. These findings provide mechanistic insight into lidocaine‐associated neurotoxicity and a preliminary theoretical basis for improving the safety evaluation of pediatric lidocaine‐containing formulations.

## Author Contributions

Suidong Ouyang and Weidong Liu proposed and supervised the project. Min Jiang, Liping Chen, Jingyi Deng, Hangyu Li, Yuqia Xie, and Xueqin Huang performed the experiments and analyzed the data. Min Jiang and Liping Chen wrote the manuscript. All authors have given approval to the final version of the manuscript.

## Funding

This work was supported by the National Natural Science Foundation of China (82370039), The Talent Development Foundation of The First Dongguan Affiliated Hospital of Guangdong Medical University (PU2024002, PU2023001, GCC2024004, GCC2023021), Guangdong Basic and Applied Basic Research Foundation (2024A1515140157), Science and Technology Special Envoy Project of Songshan Lake District of Dongguan City (20234404‐01KCJG).

## Ethics Statement

The authors have nothing to report.

## Consent

The authors have nothing to report.

## Conflicts of Interest

The authors declare no conflicts of interest.

## Supporting information


**Figure S1:** Molecular interaction modeling supports stable lidocaine binding to AKT1/PTGS2.
**Table S1:** Specific parameters of hub genes for lidocaine‐epilepsy.

## Data Availability

The data that support the findings of this study are available on request from the corresponding author. The data are not publicly available due to privacy or ethical restrictions.
