# Supplementary material for: Systems Toxicology Indicates AKT1/PTGS2‐Centered Neuroinflammatory‐Mitochondrial Crosstalk in Lidocaine Associated Neurotoxicity
Source: CNS Neurosci Ther. 2026 Oct 26;32(10):e71218. doi: 10.1002/cns.71218 (PMC13696985; doi:10.1002/cns.71218)
Supplement: Supplementary file 1 [file CNS-32-e71218-s001.docx]

**Systems Toxicology Indicates AKT1/PTGS2-Centered Neuroinflammatory-Mitochondrial Crosstalk in Lidocaine Associated Neurotoxicity**

**Figure S1 Molecular interaction modeling supports stable lidocaine binding to AKT1/PTGS2**

**Table S1 Specific parameters of hub genes for lidocaine-epilepsy**

**Figure S1. Molecular interaction modeling supports stable lidocaine binding to AKT1/PTGS2**

(A–F) CytoHubba-based hub gene identification using six topological algorithms: MNC, Degree, BottleNeck, Betweenness, Closeness, and Radiality. Node size reflects network centrality. (G) Venn diagram of the top-ranked genes identified across the six algorithms, highlighting consistently enriched hub genes associated with lidocaine-related neurotoxicity.

**Table S1 Specific parameters of hub genes for lidocaine-epilepsy**

| Gene | MNC | Degree | BottleNeck | Betweenness | Closeness | Radiality |
| --- | --- | --- | --- | --- | --- | --- |
| AKT1 | 7 | 10 | 9 | 85.33333 | 11.83333 | 4.0000 |
| PTGS2 | 5 | 7 | 7 | 64.66667 | 10.50000 | 3.85714 |
| FOS | 6 | 6 | 3 | 10.83333 | 9.83333 | 3.71429 |
| NFE2L2 | 3 | 4 | 2 | 26.00000 | 8.83333 | 3.57143 |
| PDGFRB | 4 | 4 | 2 | 4.50000 | 8.66667 | 3.50000 |
| LRRK2 | 2 | 3 | 1 | 6.00000 | 7.66667 | 3.21429 |
| PRKCB | 2 | 2 | 1 | 0 | 7.25000 | 3.14286 |
